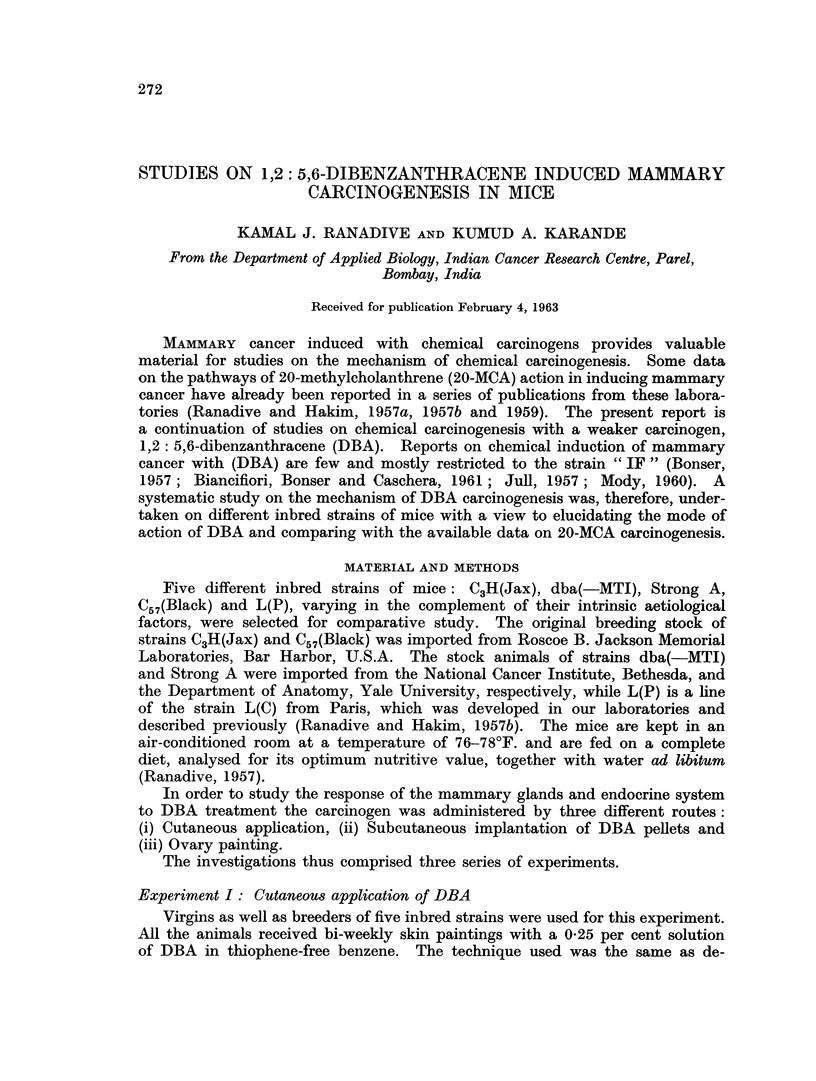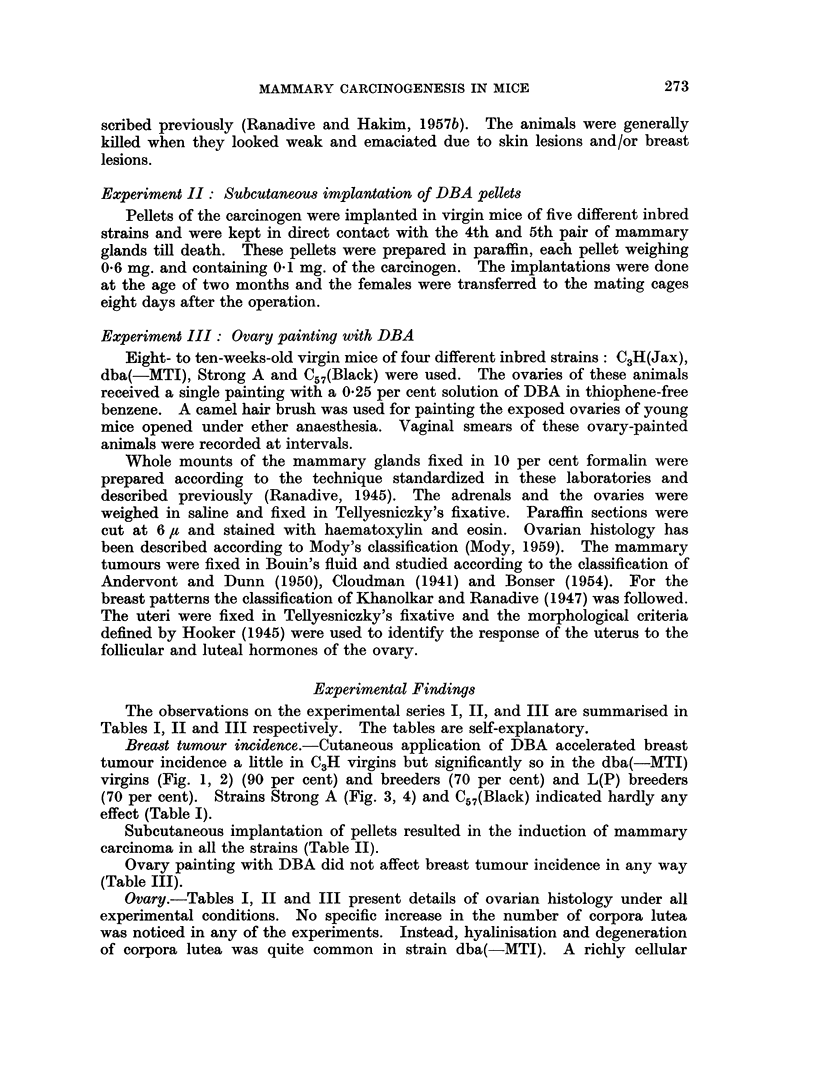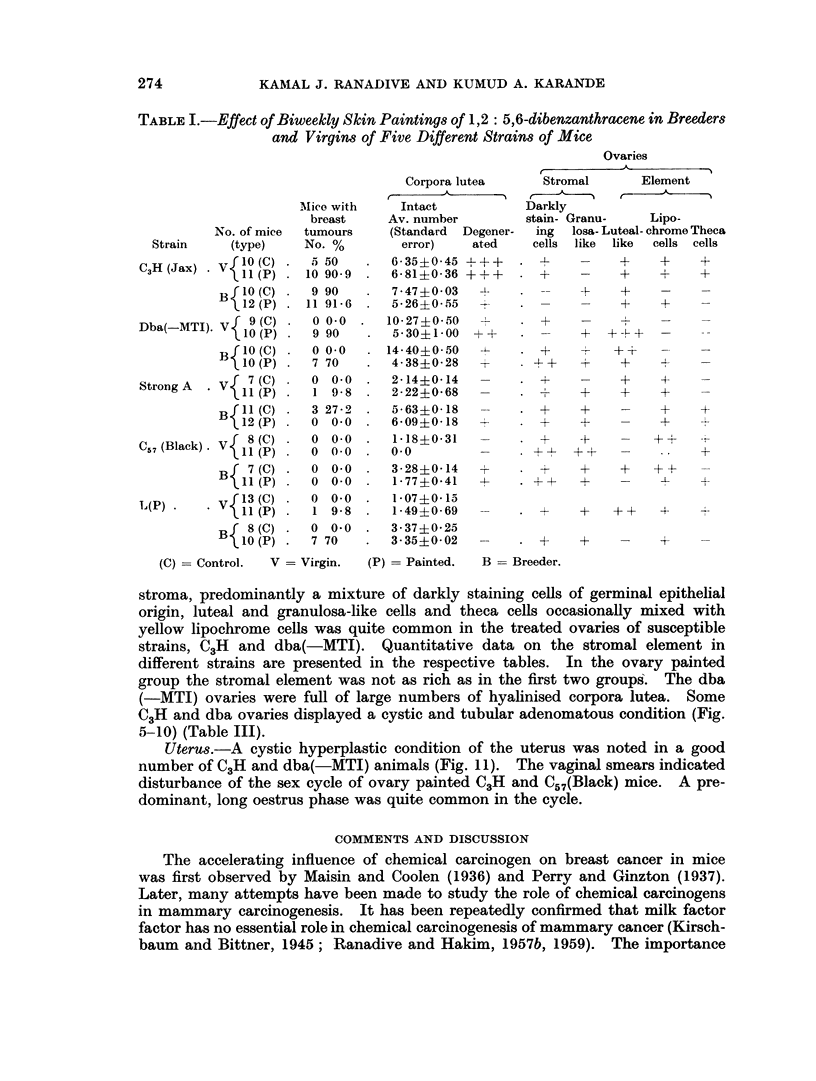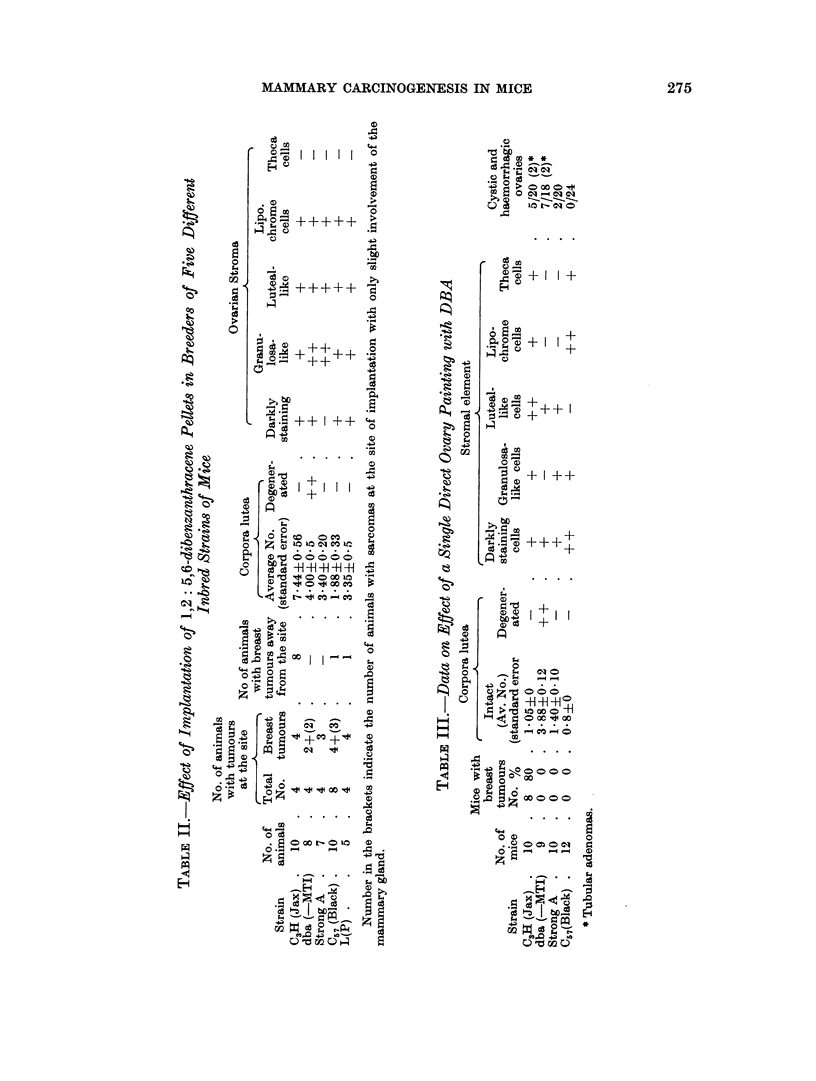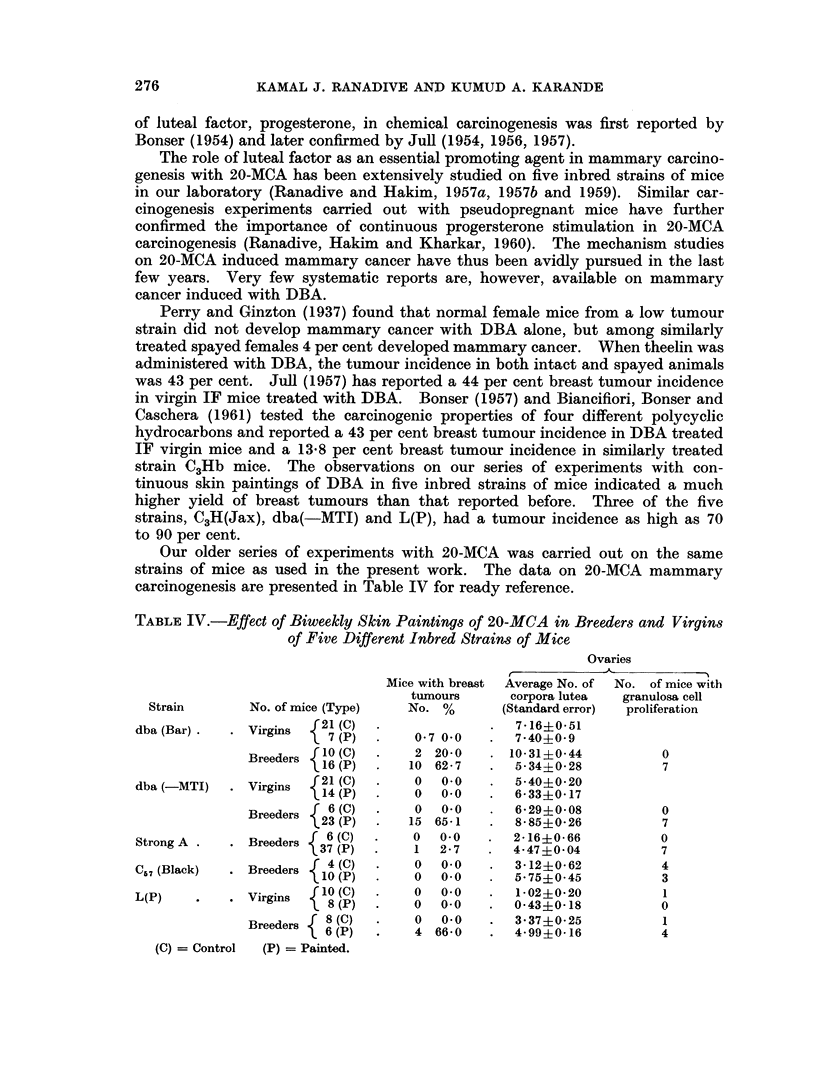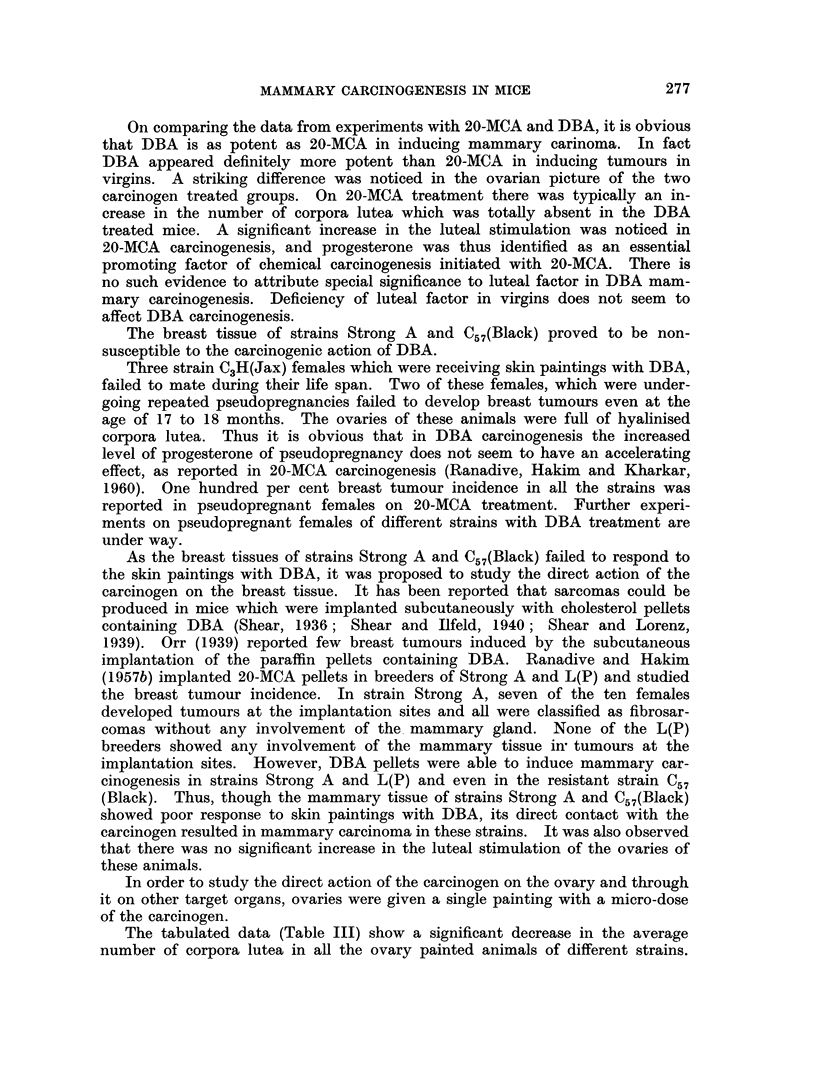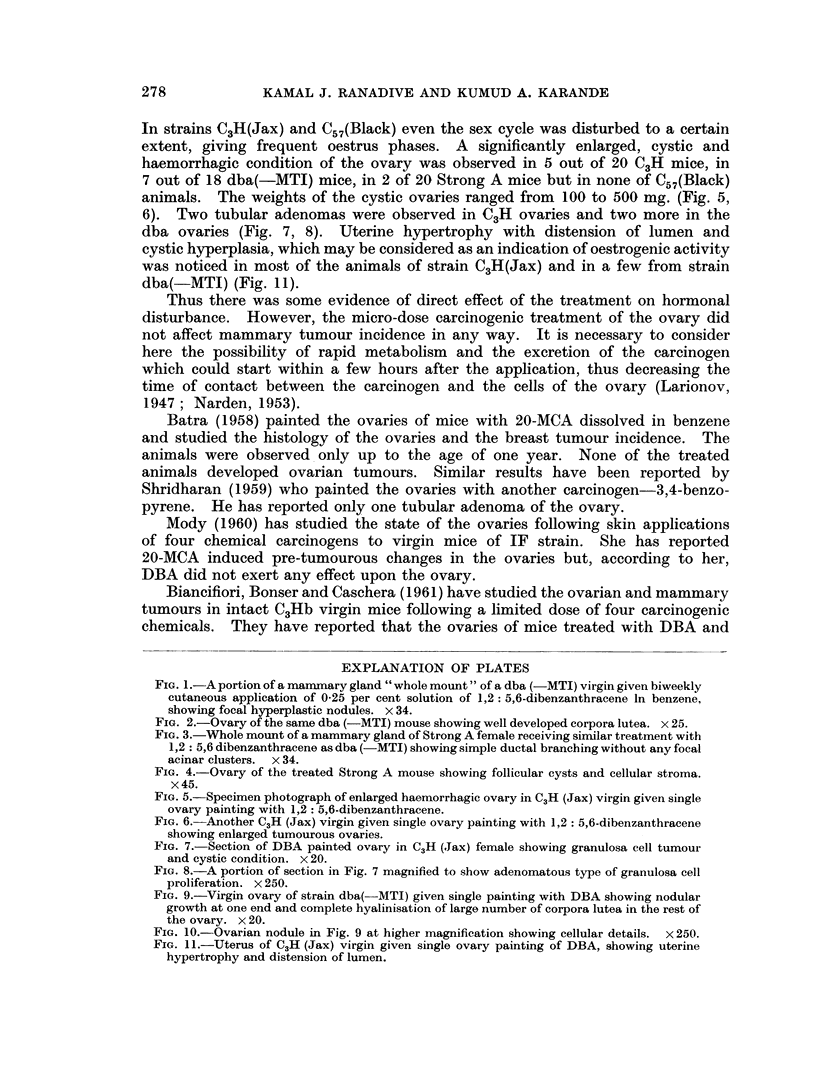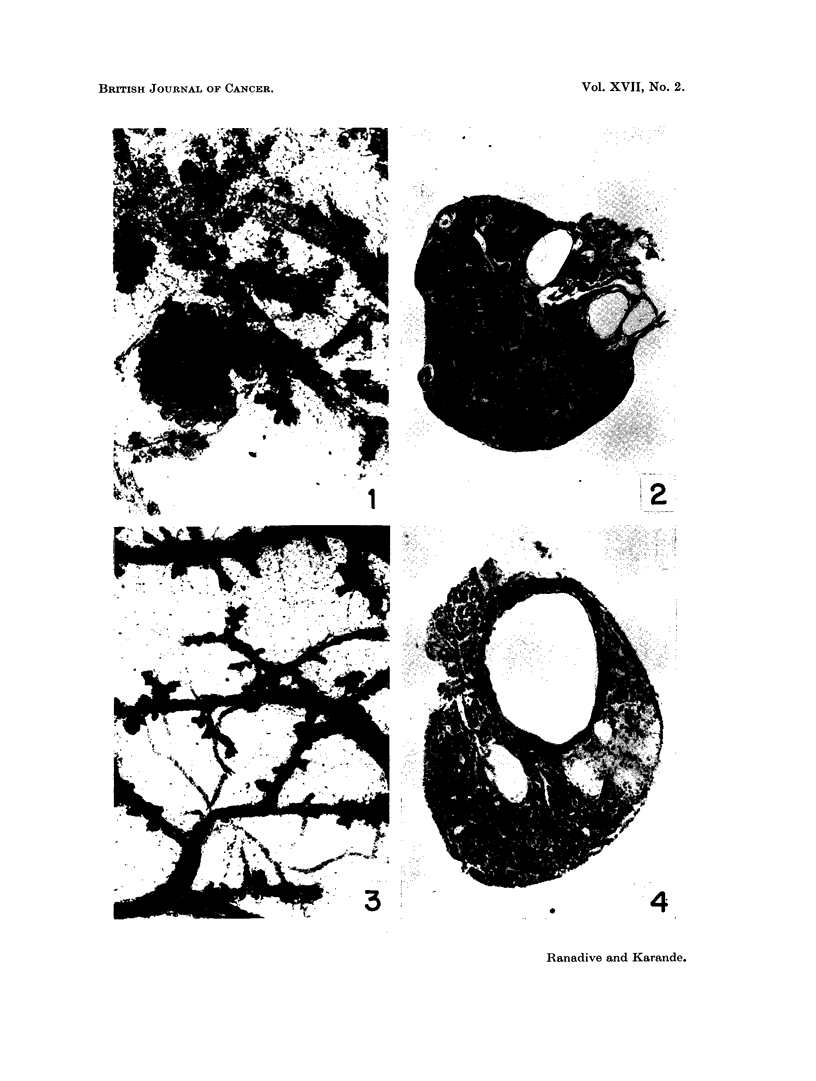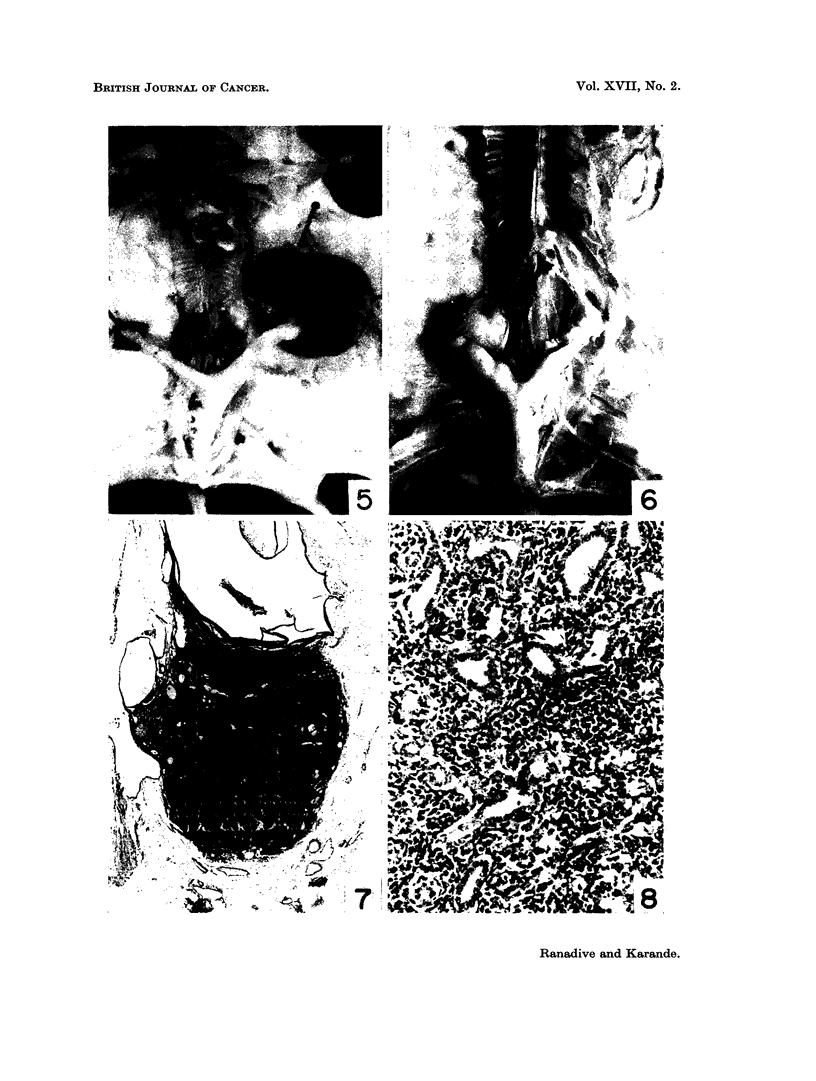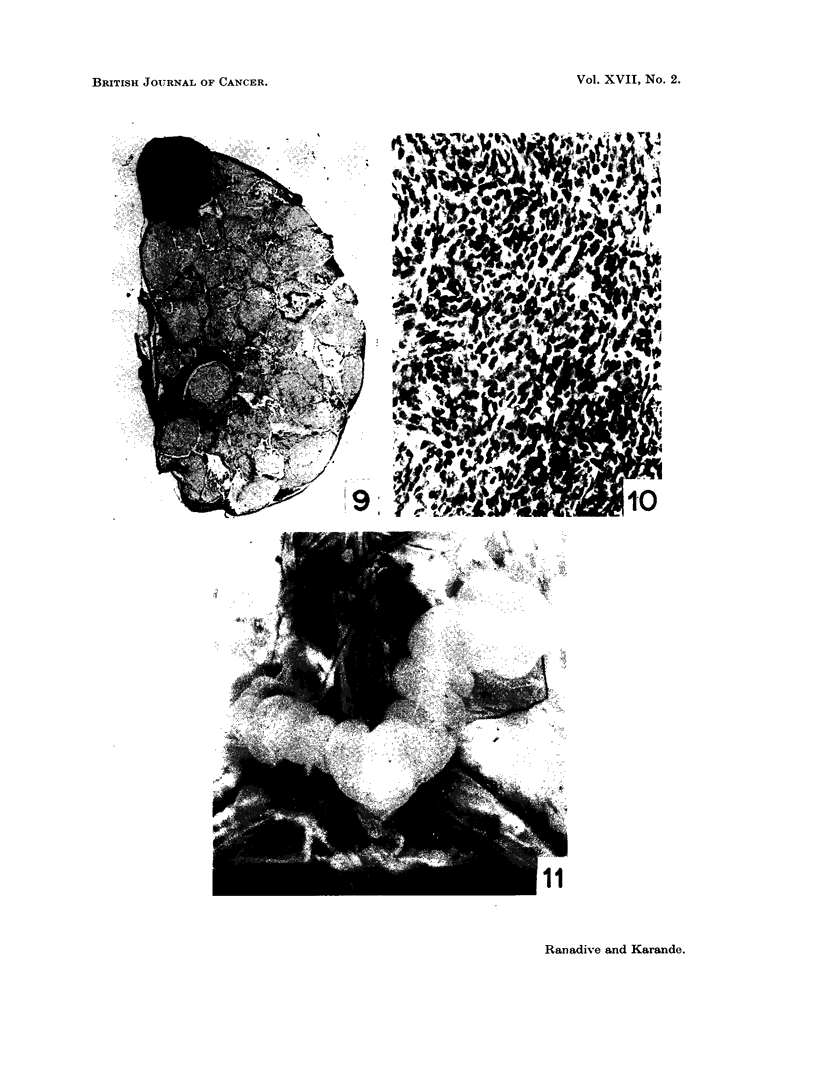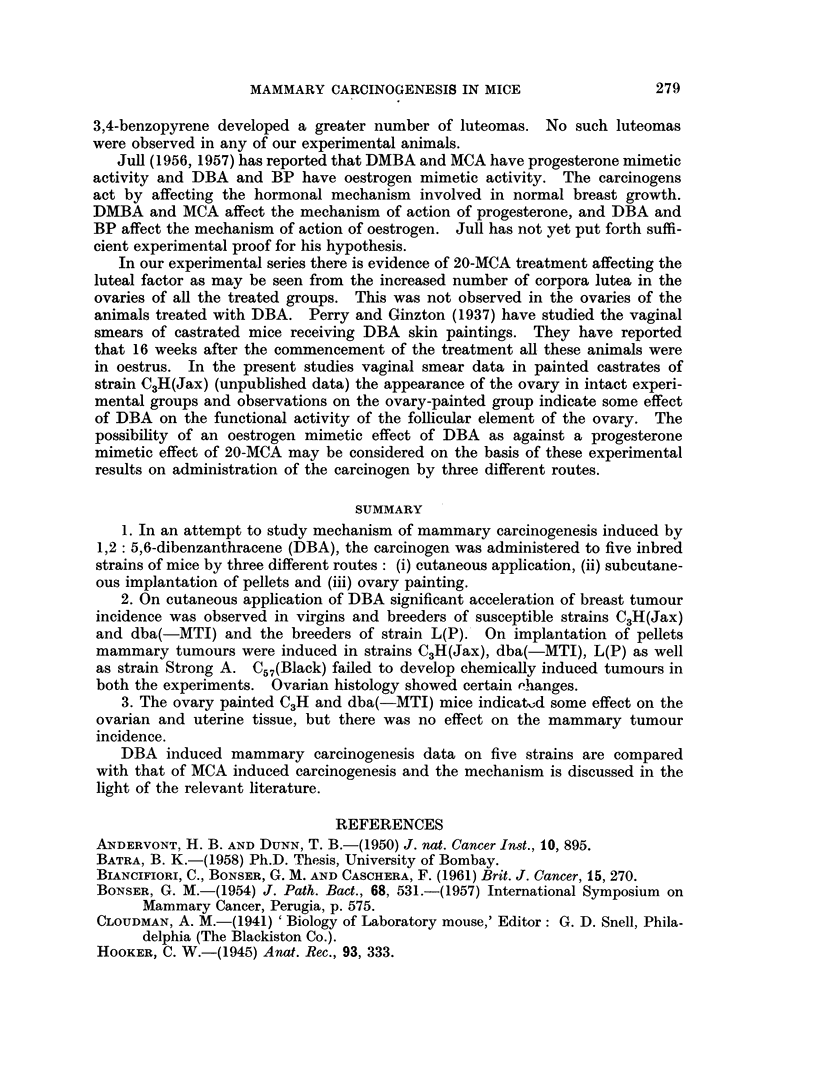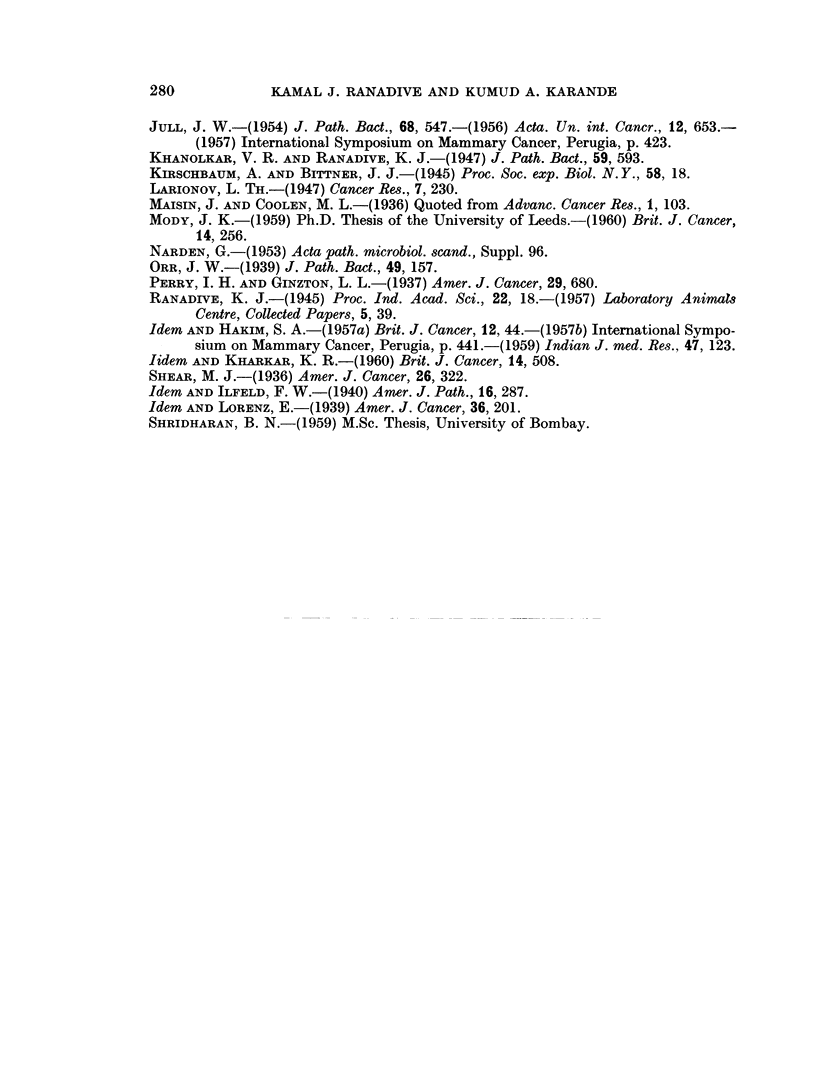# Studies on 1,2:5,6-Dibenzanthracene Induced Mammary Carcinogenesis in Mice

**DOI:** 10.1038/bjc.1963.39

**Published:** 1963-06

**Authors:** Kamal J. Ranadive, Kumud A. Karande

## Abstract

**Images:**


### STUDIES ON 1,2 : 5,6-DIBENZANTHRACENE INDUCED MAMMARY CARCINOGENESIS IN MICE

KAMAL J. RANADIVE AND KUMUD A. KARANDE

*From the Department of Applied Biology, Indian Cancer Research Centre, Parel, Bombay, India*

Received for publication February 4, 1963

MAMMARY cancer induced with chemical carcinogens provides valuable material for studies on the mechanism of chemical carcinogenesis. Some data on the pathways of 20-methylcholanthrene (20-MCA) action in inducing mammary cancer have already been reported in a series of publications from these laboratories (Ranadive and Hakim, 1957*a*, 1957*b* and 1959). The present report is a continuation of studies on chemical carcinogenesis with a weaker carcinogen, 1,2 : 5,6-dibenzanthracene (DBA). Reports on chemical induction of mammary cancer with (DBA) are few and mostly restricted to the strain " IF " (Bonser, 1957 ; Biancifiori, Bonser and Caschera, 1961 ; Jull, 1957 ; Mody, 1960). A systematic study on the mechanism of DBA carcinogenesis was, therefore, undertaken on different inbred strains of mice with a view to elucidating the mode of action of DBA and comparing with the available data on 20-MCA carcinogenesis.

### MATERIAL AND METHODS

Five different inbred strains of mice : $C_3H$(Jax), dba(—MTI), Strong A, $C_{57}$(Black) and L(P), varying in the complement of their intrinsic aetiological factors, were selected for comparative study. The original breeding stock of strains $C_3H$(Jax) and $C_{57}$(Black) was imported from Roscoe B. Jackson Memorial Laboratories, Bar Harbor, U.S.A. The stock animals of strains dba(—MTI) and Strong A were imported from the National Cancer Institute, Bethesda, and the Department of Anatomy, Yale University, respectively, while L(P) is a line of the strain L(C) from Paris, which was developed in our laboratories and described previously (Ranadive and Hakim, 1957*b*). The mice are kept in an air-conditioned room at a temperature of 76–78°F. and are fed on a complete diet, analysed for its optimum nutritive value, together with water *ad libitum* (Ranadive, 1957).

In order to study the response of the mammary glands and endocrine system to DBA treatment the carcinogen was administered by three different routes : (i) Cutaneous application, (ii) Subcutaneous implantation of DBA pellets and (iii) Ovary painting.

The investigations thus comprised three series of experiments.

### *Experiment I : Cutaneous application of DBA*

Virgins as well as breeders of five inbred strains were used for this experiment. All the animals received bi-weekly skin paintings with a 0·25 per cent solution of DBA in thiophene-free benzene. The technique used was the same as described previously (Ranadive and Hakim, 1957*b*). The animals were generally killed when they looked weak and emaciated due to skin lesions and/or breast lesions.

*Experiment II : Subcutaneous implantation of DBA pellets*

Pellets of the carcinogen were implanted in virgin mice of five different inbred strains and were kept in direct contact with the 4th and 5th pair of mammary glands till death. These pellets were prepared in paraffin, each pellet weighing 0·6 mg. and containing 0·1 mg. of the carcinogen. The implantations were done at the age of two months and the females were transferred to the mating cages eight days after the operation.

*Experiment III : Ovary painting with DBA*

Eight- to ten-weeks-old virgin mice of four different inbred strains : $C_3H$(Jax), dba(—MTI), Strong A and $C_{57}$(Black) were used. The ovaries of these animals received a single painting with a 0·25 per cent solution of DBA in thiophene-free benzene. A camel hair brush was used for painting the exposed ovaries of young mice opened under ether anaesthesia. Vaginal smears of these ovary-painted animals were recorded at intervals.

Whole mounts of the mammary glands fixed in 10 per cent formalin were prepared according to the technique standardized in these laboratories and described previously (Ranadive, 1945). The adrenals and the ovaries were weighed in saline and fixed in Tellyesniczky's fixative. Paraffin sections were cut at 6 $\mu$ and stained with haematoxylin and eosin. Ovarian histology has been described according to Mody's classification (Mody, 1959). The mammary tumours were fixed in Bouin's fluid and studied according to the classification of Andervont and Dunn (1950), Cloudman (1941) and Bonser (1954). For the breast patterns the classification of Khanolkar and Ranadive (1947) was followed. The uteri were fixed in Tellyesniczky's fixative and the morphological criteria defined by Hooker (1945) were used to identify the response of the uterus to the follicular and luteal hormones of the ovary.

### *Experimental Findings*

The observations on the experimental series I, II, and III are summarised in Tables I, II and III respectively. The tables are self-explanatory.

*Breast tumour incidence.*—Cutaneous application of DBA accelerated breast tumour incidence a little in $C_3H$ virgins but significantly so in the dba(—MTI) virgins (Fig. 1, 2) (90 per cent) and breeders (70 per cent) and L(P) breeders (70 per cent). Strains Strong A (Fig. 3, 4) and $C_{57}$(Black) indicated hardly any effect (Table I).

Subcutaneous implantation of pellets resulted in the induction of mammary carcinoma in all the strains (Table II).

Ovary painting with DBA did not affect breast tumour incidence in any way (Table III).

*Ovary.*—Tables I, II and III present details of ovarian histology under all experimental conditions. No specific increase in the number of corpora lutea was noticed in any of the experiments. Instead, hyalinisation and degeneration of corpora lutea was quite common in strain dba(—MTI). A richly cellular

TABLE I.—*Effect of Biweekly Skin Paintings of* 1,2 : 5,6-*dibenzanthracene in Breeders and Virgins of Five Different Strains of Mice*

| Strain | No. of mice (type) | Mice with breast tumours No. | Mice with breast tumours % | Corpora lutea: Intact Av. number (Standard error) | Corpora lutea: Degenerated | Ovaries, Stromal: Darkly staining cells | Ovaries, Stromal: Granulosa-like | Ovaries, Element: Luteal-like | Ovaries, Element: Lipochrome cells | Ovaries, Element: Theca cells |
|---|---|---|---|---|---|---|---|---|---|---|
| $C_3H$ (Jax) | V 10 (C) | 5 | 50 | 6·35±0·45 | +++ | + | − | + | + | + |
| | V 11 (P) | 10 | 90·9 | 6·81±0·36 | +++ | + | − | + | + | + |
| | B 10 (C) | 9 | 90 | 7·47±0·03 | + | − | + | + | − | − |
| | B 12 (P) | 11 | 91·6 | 5·26±0·55 | + | − | − | + | + | − |
| Dba(—MTI) | V 9 (C) | 0 | 0·0 | 10·27±0·50 | + | + | − | + | − | − |
| | V 10 (P) | 9 | 90 | 5·30±1·00 | ++ | − | + | +++ | − | − |
| | B 10 (C) | 0 | 0·0 | 14·40±0·50 | + | + | + | ++ | − | − |
| | B 10 (P) | 7 | 70 | 4·38±0·28 | + | ++ | + | + | + | − |
| Strong A | V 7 (C) | 0 | 0·0 | 2·14±0·14 | − | + | − | + | + | − |
| | V 11 (P) | 1 | 9·8 | 2·22±0·68 | − | + | + | + | + | − |
| | B 11 (C) | 3 | 27·2 | 5·63±0·18 | − | + | + | − | + | + |
| | B 12 (P) | 0 | 0·0 | 6·09±0·18 | + | + | + | − | + | + |
| $C_{57}$ (Black) | V 8 (C) | 0 | 0·0 | 1·18±0·31 | − | + | + | − | ++ | + |
| | V 11 (P) | 0 | 0·0 | 0·0 | − | ++ | ++ | − | .. | + |
| | B 7 (C) | 0 | 0·0 | 3·28±0·14 | + | + | + | + | ++ | − |
| | B 11 (P) | 0 | 0·0 | 1·77±0·41 | + | ++ | + | − | + | + |
| L(P) | V 13 (C) | 0 | 0·0 | 1·07±0·15 | | | | | | |
| | V 11 (P) | 1 | 9·8 | 1·49±0·69 | − | + | + | ++ | + | + |
| | B 8 (C) | 0 | 0·0 | 3·37±0·25 | | | | | | |
| | B 10 (P) | 7 | 70 | 3·35±0·02 | − | + | + | − | + | − |

(C) = Control. V = Virgin. (P) = Painted. B = Breeder.

stroma, predominantly a mixture of darkly staining cells of germinal epithelial origin, luteal and granulosa-like cells and theca cells occasionally mixed with yellow lipochrome cells was quite common in the treated ovaries of susceptible strains, $C_3H$ and dba(—MTI). Quantitative data on the stromal element in different strains are presented in the respective tables. In the ovary painted group the stromal element was not as rich as in the first two groups. The dba (—MTI) ovaries were full of large numbers of hyalinised corpora lutea. Some $C_3H$ and dba ovaries displayed a cystic and tubular adenomatous condition (Fig. 5–10) (Table III).

*Uterus.*—A cystic hyperplastic condition of the uterus was noted in a good number of $C_3H$ and dba(—MTI) animals (Fig. 11). The vaginal smears indicated disturbance of the sex cycle of ovary painted $C_3H$ and $C_{57}$(Black) mice. A predominant, long oestrus phase was quite common in the cycle.

### COMMENTS AND DISCUSSION

The accelerating influence of chemical carcinogen on breast cancer in mice was first observed by Maisin and Coolen (1936) and Perry and Ginzton (1937). Later, many attempts have been made to study the role of chemical carcinogens in mammary carcinogenesis. It has been repeatedly confirmed that milk factor factor has no essential role in chemical carcinogenesis of mammary cancer (Kirschbaum and Bittner, 1945 ; Ranadive and Hakim, 1957*b*, 1959). The importance

TABLE II.—*Effect of Implantation of* 1,2 : 5,6-*dibenzanthracene Pellets in Breeders of Five Different Inbred Strains of Mice*

| Strain | No. of animals | No. of animals with tumours at the site: Total No. | No. of animals with tumours at the site: Breast tumours | No of animals with breast tumours away from the site | Corpora lutea: Average No. (standard error) | Corpora lutea: Degenerated | Ovarian Stroma: Darkly staining | Ovarian Stroma: Granulosa-like | Ovarian Stroma: Luteal-like | Ovarian Stroma: Lipochrome cells | Ovarian Stroma: Theca cells |
|---|---|---|---|---|---|---|---|---|---|---|---|
| $C_3H$ (Jax) | 10 | 4 | 4 | 8 | 7·44±0·56 | − | + | + | + | + | − |
| dba (—MTI) | 8 | 4 | 2+(2) | — | 4·00±0·5 | ++ | + | ++ | + | + | − |
| Strong A | 7 | 4 | 3 | — | 3·40±0·20 | − | − | ++ | + | + | − |
| $C_{57}$ (Black) | 10 | 8 | 4+(3) | 1 | 1·88±0·33 | − | + | + | + | + | − |
| L(P) | 5 | 4 | 4 | 1 | 3·35±0·5 | − | + | + | + | + | − |

Number in the brackets indicate the number of animals with sarcomas at the site of implantation with only slight involvement of the mammary gland.

TABLE III.—*Data on Effect of a Single Direct Ovary Painting with DBA*

| Strain | No. of mice | Mice with breast tumours No. | Mice with breast tumours % | Corpora lutea: Intact (Av. No.) (standard error | Corpora lutea: Degenerated | Stromal element: Darkly staining cells | Stromal element: Granulosa-like cells | Stromal element: Luteal-like cells | Stromal element: Lipochrome cells | Stromal element: Theca cells | Cystic and haemorrhagic ovaries |
|---|---|---|---|---|---|---|---|---|---|---|---|
| $C_3H$ (Jax) | 10 | 8 | 80 | 1·05±0 | − | + | + | ++ | + | + | 5/20 (2)* |
| dba (—MTI) | 9 | 0 | 0 | 3·88±0·12 | ++ | + | − | + | − | − | 7/18 (2)* |
| Strong A | 10 | 0 | 0 | 1·40±0·10 | − | + | + | + | − | − | 2/20 |
| $C_{57}$(Black) | 12 | 0 | 0 | 0·8±0 | − | ++ | + | − | ++ | + | 0/24 |

* Tubular adenomas.

of luteal factor, progesterone, in chemical carcinogenesis was first reported by Bonser (1954) and later confirmed by Jull (1954, 1956, 1957).

The role of luteal factor as an essential promoting agent in mammary carcinogenesis with 20-MCA has been extensively studied on five inbred strains of mice in our laboratory (Ranadive and Hakim, 1957*a*, 1957*b* and 1959). Similar carcinogenesis experiments carried out with pseudopregnant mice have further confirmed the importance of continuous progersterone stimulation in 20-MCA carcinogenesis (Ranadive, Hakim and Kharkar, 1960). The mechanism studies on 20-MCA induced mammary cancer have thus been avidly pursued in the last few years. Very few systematic reports are, however, available on mammary cancer induced with DBA.

Perry and Ginzton (1937) found that normal female mice from a low tumour strain did not develop mammary cancer with DBA alone, but among similarly treated spayed females 4 per cent developed mammary cancer. When theelin was administered with DBA, the tumour incidence in both intact and spayed animals was 43 per cent. Jull (1957) has reported a 44 per cent breast tumour incidence in virgin IF mice treated with DBA. Bonser (1957) and Biancifiori, Bonser and Caschera (1961) tested the carcinogenic properties of four different polycyclic hydrocarbons and reported a 43 per cent breast tumour incidence in DBA treated IF virgin mice and a 13·8 per cent breast tumour incidence in similarly treated strain $C_3Hb$ mice. The observations on our series of experiments with continuous skin paintings of DBA in five inbred strains of mice indicated a much higher yield of breast tumours than that reported before. Three of the five strains, $C_3H$(Jax), dba(—MTI) and L(P), had a tumour incidence as high as 70 to 90 per cent.

Our older series of experiments with 20-MCA was carried out on the same strains of mice as used in the present work. The data on 20-MCA mammary carcinogenesis are presented in Table IV for ready reference.

TABLE IV.—*Effect of Biweekly Skin Paintings of 20-MCA in Breeders and Virgins of Five Different Inbred Strains of Mice*

| Strain | No. of mice (Type) | | Mice with breast tumours No. | % | Ovaries: Average No. of corpora lutea (Standard error) | Ovaries: No. of mice with granulosa cell proliferation |
|---|---|---|---|---|---|---|
| dba (Bar) | Virgins | 21 (C) | | | 7·16±0·51 | |
| | | 7 (P) | 0·7 | 0·0 | 7·40±0·9 | |
| | Breeders | 10 (C) | 2 | 20·0 | 10·31±0·44 | 0 |
| | | 16 (P) | 10 | 62·7 | 5·34±0·28 | 7 |
| dba (—MTI) | Virgins | 21 (C) | 0 | 0·0 | 5·40±0·20 | |
| | | 14 (P) | 0 | 0·0 | 6·33±0·17 | |
| | Breeders | 6 (C) | 0 | 0·0 | 6·29±0·08 | 0 |
| | | 23 (P) | 15 | 65·1 | 8·85±0·26 | 7 |
| Strong A | Breeders | 6 (C) | 0 | 0·0 | 2·16±0·66 | 0 |
| | | 37 (P) | 1 | 2·7 | 4·47±0·04 | 7 |
| $C_{57}$ (Black) | Breeders | 4 (C) | 0 | 0·0 | 3·12±0·62 | 4 |
| | | 10 (P) | 0 | 0·0 | 5·75±0·45 | 3 |
| L(P) | Virgins | 10 (C) | 0 | 0·0 | 1·02±0·20 | 1 |
| | | 8 (P) | 0 | 0·0 | 0·43±0·18 | 0 |
| | Breeders | 8 (C) | 0 | 0·0 | 3·37±0·25 | 1 |
| | | 6 (P) | 4 | 66·0 | 4·99±0·16 | 4 |

(C) = Control (P) = Painted.

On comparing the data from experiments with 20-MCA and DBA, it is obvious that DBA is as potent as 20-MCA in inducing mammary carinoma. In fact DBA appeared definitely more potent than 20-MCA in inducing tumours in virgins. A striking difference was noticed in the ovarian picture of the two carcinogen treated groups. On 20-MCA treatment there was typically an increase in the number of corpora lutea which was totally absent in the DBA treated mice. A significant increase in the luteal stimulation was noticed in 20-MCA carcinogenesis, and progesterone was thus identified as an essential promoting factor of chemical carcinogenesis initiated with 20-MCA. There is no such evidence to attribute special significance to luteal factor in DBA mammary carcinogenesis. Deficiency of luteal factor in virgins does not seem to affect DBA carcinogenesis.

The breast tissue of strains Strong A and $C_{57}$(Black) proved to be non-susceptible to the carcinogenic action of DBA.

Three strain $C_3H$(Jax) females which were receiving skin paintings with DBA, failed to mate during their life span. Two of these females, which were undergoing repeated pseudopregnancies failed to develop breast tumours even at the age of 17 to 18 months. The ovaries of these animals were full of hyalinised corpora lutea. Thus it is obvious that in DBA carcinogenesis the increased level of progesterone of pseudopregnancy does not seem to have an accelerating effect, as reported in 20-MCA carcinogenesis (Ranadive, Hakim and Kharkar, 1960). One hundred per cent breast tumour incidence in all the strains was reported in pseudopregnant females on 20-MCA treatment. Further experiments on pseudopregnant females of different strains with DBA treatment are under way.

As the breast tissues of strains Strong A and $C_{57}$(Black) failed to respond to the skin paintings with DBA, it was proposed to study the direct action of the carcinogen on the breast tissue. It has been reported that sarcomas could be produced in mice which were implanted subcutaneously with cholesterol pellets containing DBA (Shear, 1936; Shear and Ilfeld, 1940; Shear and Lorenz, 1939). Orr (1939) reported few breast tumours induced by the subcutaneous implantation of the paraffin pellets containing DBA. Ranadive and Hakim (1957*b*) implanted 20-MCA pellets in breeders of Strong A and L(P) and studied the breast tumour incidence. In strain Strong A, seven of the ten females developed tumours at the implantation sites and all were classified as fibrosarcomas without any involvement of the mammary gland. None of the L(P) breeders showed any involvement of the mammary tissue in tumours at the implantation sites. However, DBA pellets were able to induce mammary carcinogenesis in strains Strong A and L(P) and even in the resistant strain $C_{57}$ (Black). Thus, though the mammary tissue of strains Strong A and $C_{57}$(Black) showed poor response to skin paintings with DBA, its direct contact with the carcinogen resulted in mammary carcinoma in these strains. It was also observed that there was no significant increase in the luteal stimulation of the ovaries of these animals.

In order to study the direct action of the carcinogen on the ovary and through it on other target organs, ovaries were given a single painting with a micro-dose of the carcinogen.

The tabulated data (Table III) show a significant decrease in the average number of corpora lutea in all the ovary painted animals of different strains.

In strains $C_3H$(Jax) and $C_{57}$(Black) even the sex cycle was disturbed to a certain extent, giving frequent oestrus phases. A significantly enlarged, cystic and haemorrhagic condition of the ovary was observed in 5 out of 20 $C_3H$ mice, in 7 out of 18 dba(—MTI) mice, in 2 of 20 Strong A mice but in none of $C_{57}$(Black) animals. The weights of the cystic ovaries ranged from 100 to 500 mg. (Fig. 5, 6). Two tubular adenomas were observed in $C_3H$ ovaries and two more in the dba ovaries (Fig. 7, 8). Uterine hypertrophy with distension of lumen and cystic hyperplasia, which may be considered as an indication of oestrogenic activity was noticed in most of the animals of strain $C_3H$(Jax) and in a few from strain dba(—MTI) (Fig. 11).

Thus there was some evidence of direct effect of the treatment on hormonal disturbance. However, the micro-dose carcinogenic treatment of the ovary did not affect mammary tumour incidence in any way. It is necessary to consider here the possibility of rapid metabolism and the excretion of the carcinogen which could start within a few hours after the application, thus decreasing the time of contact between the carcinogen and the cells of the ovary (Larionov, 1947 ; Narden, 1953).

Batra (1958) painted the ovaries of mice with 20-MCA dissolved in benzene and studied the histology of the ovaries and the breast tumour incidence. The animals were observed only up to the age of one year. None of the treated animals developed ovarian tumours. Similar results have been reported by Shridharan (1959) who painted the ovaries with another carcinogen—3,4-benzopyrene. He has reported only one tubular adenoma of the ovary.

Mody (1960) has studied the state of the ovaries following skin applications of four chemical carcinogens to virgin mice of IF strain. She has reported 20-MCA induced pre-tumourous changes in the ovaries but, according to her, DBA did not exert any effect upon the ovary.

Biancifiori, Bonser and Caschera (1961) have studied the ovarian and mammary tumours in intact $C_3Hb$ virgin mice following a limited dose of four carcinogenic chemicals. They have reported that the ovaries of mice treated with DBA and

---

EXPLANATION OF PLATES

FIG. 1.—A portion of a mammary gland "whole mount" of a dba (—MTI) virgin given biweekly cutaneous application of 0·25 per cent solution of 1,2 : 5,6-dibenzanthracene ln benzene, showing focal hyperplastic nodules. ×34.

FIG. 2.—Ovary of the same dba (—MTI) mouse showing well developed corpora lutea. ×25.

FIG. 3.—Whole mount of a mammary gland of Strong A female receiving similar treatment with 1,2 : 5,6 dibenzanthracene as dba (—MTI) showing simple ductal branching without any focal acinar clusters. ×34.

FIG. 4.—Ovary of the treated Strong A mouse showing follicular cysts and cellular stroma. ×45.

FIG. 5.—Specimen photograph of enlarged haemorrhagic ovary in $C_3H$ (Jax) virgin given single ovary painting with 1,2 : 5,6-dibenzanthracene.

FIG. 6.—Another $C_3H$ (Jax) virgin given single ovary painting with 1,2 : 5,6-dibenzanthracene showing enlarged tumourous ovaries.

FIG. 7.—Section of DBA painted ovary in $C_3H$ (Jax) female showing granulosa cell tumour and cystic condition. ×20.

FIG. 8.—A portion of section in Fig. 7 magnified to show adenomatous type of granulosa cell proliferation. ×250.

FIG. 9.—Virgin ovary of strain dba(—MTI) given single painting with DBA showing nodular growth at one end and complete hyalinisation of large number of corpora lutea in the rest of the ovary. ×20.

FIG. 10.—Ovarian nodule in Fig. 9 at higher magnification showing cellular details. ×250.

FIG. 11.—Uterus of $C_3H$ (Jax) virgin given single ovary painting of DBA, showing uterine hypertrophy and distension of lumen.

Ranadive and Karande.

5

6

7

8

9

10

11

3,4-benzopyrene developed a greater number of luteomas. No such luteomas were observed in any of our experimental animals.

Jull (1956, 1957) has reported that DMBA and MCA have progesterone mimetic activity and DBA and BP have oestrogen mimetic activity. The carcinogens act by affecting the hormonal mechanism involved in normal breast growth. DMBA and MCA affect the mechanism of action of progesterone, and DBA and BP affect the mechanism of action of oestrogen. Jull has not yet put forth sufficient experimental proof for his hypothesis.

In our experimental series there is evidence of 20-MCA treatment affecting the luteal factor as may be seen from the increased number of corpora lutea in the ovaries of all the treated groups. This was not observed in the ovaries of the animals treated with DBA. Perry and Ginzton (1937) have studied the vaginal smears of castrated mice receiving DBA skin paintings. They have reported that 16 weeks after the commencement of the treatment all these animals were in oestrus. In the present studies vaginal smear data in painted castrates of strain $C_3H$(Jax) (unpublished data) the appearance of the ovary in intact experimental groups and observations on the ovary-painted group indicate some effect of DBA on the functional activity of the follicular element of the ovary. The possibility of an oestrogen mimetic effect of DBA as against a progesterone mimetic effect of 20-MCA may be considered on the basis of these experimental results on administration of the carcinogen by three different routes.

### SUMMARY

1. In an attempt to study mechanism of mammary carcinogenesis induced by 1,2 : 5,6-dibenzanthracene (DBA), the carcinogen was administered to five inbred strains of mice by three different routes : (i) cutaneous application, (ii) subcutaneous implantation of pellets and (iii) ovary painting.

2. On cutaneous application of DBA significant acceleration of breast tumour incidence was observed in virgins and breeders of susceptible strains $C_3H$(Jax) and dba(—MTI) and the breeders of strain L(P). On implantation of pellets mammary tumours were induced in strains $C_3H$(Jax), dba(—MTI), L(P) as well as strain Strong A. $C_{57}$(Black) failed to develop chemically induced tumours in both the experiments. Ovarian histology showed certain changes.

3. The ovary painted $C_3H$ and dba(—MTI) mice indicated some effect on the ovarian and uterine tissue, but there was no effect on the mammary tumour incidence.

DBA induced mammary carcinogenesis data on five strains are compared with that of MCA induced carcinogenesis and the mechanism is discussed in the light of the relevant literature.

### REFERENCES

ANDERVONT, H. B. AND DUNN, T. B.—(1950) *J. nat. Cancer Inst.*, **10**, 895.

BATRA, B. K.—(1958) Ph.D. Thesis, University of Bombay.

BIANCIFIORI, C., BONSER, G. M. AND CASCHERA, F. (1961) *Brit. J. Cancer*, **15**, 270.

BONSER, G. M.—(1954) *J. Path. Bact.*, **68**, 531.—(1957) International Symposium on Mammary Cancer, Perugia, p. 575.

CLOUDMAN, A. M.—(1941) 'Biology of Laboratory mouse,' Editor : G. D. Snell, Philadelphia (The Blackiston Co.).

HOOKER, C. W.—(1945) *Anat. Rec.*, **93**, 333.

JULL, J. W.—(1954) *J. Path. Bact.*, **68**, 547.—(1956) *Acta. Un. int. Cancr.*, **12**, 653.—(1957) International Symposium on Mammary Cancer, Perugia, p. 423.
KHANOLKAR, V. R. AND RANADIVE, K. J.—(1947) *J. Path. Bact.*, **59**, 593.
KIRSCHBAUM, A. AND BITTNER, J. J.—(1945) *Proc. Soc. exp. Biol. N.Y.*, **58**, 18.
LARIONOV, L. TH.—(1947) *Cancer Res.*, **7**, 230.
MAISIN, J. AND COOLEN, M. L.—(1936) Quoted from *Advanc. Cancer Res.*, **1**, 103.
MODY, J. K.—(1959) Ph.D. Thesis of the University of Leeds.—(1960) *Brit. J. Cancer*, **14**, 256.
NARDEN, G.—(1953) *Acta path. microbiol. scand.*, Suppl. 96.
ORR, J. W.—(1939) *J. Path. Bact.*, **49**, 157.
PERRY, I. H. AND GINZTON, L. L.—(1937) *Amer. J. Cancer*, **29**, 680.
RANADIVE, K. J.—(1945) *Proc. Ind. Acad. Sci.*, **22**, 18.—(1957) *Laboratory Animals Centre, Collected Papers*, **5**, 39.
*Idem* AND HAKIM, S. A.—(1957*a*) *Brit. J. Cancer*, **12**, 44.—(1957*b*) International Symposium on Mammary Cancer, Perugia, p. 441.—(1959) *Indian J. med. Res.*, **47**, 123.
*Iidem* AND KHARKAR, K. R.—(1960) *Brit. J. Cancer*, **14**, 508.
SHEAR, M. J.—(1936) *Amer. J. Cancer*, **26**, 322.
*Idem* AND ILFELD, F. W.—(1940) *Amer. J. Path.*, **16**, 287.
*Idem* AND LORENZ, E.—(1939) *Amer. J. Cancer*, **36**, 201.
SHRIDHARAN, B. N.—(1959) M.Sc. Thesis, University of Bombay.